# Relationships between beliefs about statins and non-adherence in inpatients from Northwestern China: a cross-sectional survey

**DOI:** 10.3389/fphar.2023.1078215

**Published:** 2023-06-09

**Authors:** Haiyan Li, Xiaoni Jia, Hui Min, Yingli Zhang, Huichuan Wang, Yuyao Zhai

**Affiliations:** ^1^ Department of Pharmacy, Xi’an People’s Hospital (Xi’an Fourth Hospital), Xi’an, China; ^2^ Department of Science and Education, Xi’an Mental Health Center, Xi’an, China; ^3^ Department of Pharmacy, Xi’an Mental Health Center, Xi’an, China; ^4^ Department of Obstetrics, Xi’an People’s Hospital (Xi’an Fourth Hospital), Xi’an, China; ^5^ Health Management Center, Xi’an People’s Hospital (Xi’an Fourth Hospital), Xi’an, China

**Keywords:** medication adherence, statin, beliefs about medicine, nurses, pharmacists, China

## Abstract

**Background:** Studies have identified patients’ beliefs about medicines as an important determinant of non-adherence. However, scant data are available on the possible association between patients’ beliefs and statin non-adherence among adult patients in China. The objectives of this study are to assess the prevalence of statin non-adherence, and to identify the factors associated with statin non-adherence, especially the association between inpatients’ beliefs about statins and non-adherence in a tertiary hospital in the Northwestern China.

**Methods:** A cross-sectional questionnaire-based survey was carried out in the department of cardiology and neurology between February and June 2022. The Beliefs about Medicine Questionnaire (BMQ) was used to assess patients’ beliefs about statins. The Adherence to Refills and Medications Scale (ARMS) was used to assess statin adherence. Logistic regression analyses were performed to identify the factors associated with statin non-adherence. Receiver operator characteristic (ROC) was conducted to assess the performance of the logistic regression model in predicting statin non-adherence.

**Results:** A total of 524 inpatients participated and finished the questionnaire, 426 (81.3%) inpatients were non-adherent to statin, and 229 (43.7%) inpatients expressed strong beliefs about the stain treatment necessity, while 246 (47.0%) inpatients expressed strong concerns about the potential negative effects. We found that the low necessity beliefs about statin (adjusted odds ratio [OR] and 95% confidence interval [CI], 1.607 [1.019, 2.532]; *p* = 0.041), prescribed rosuvastatin (adjusted OR 1.820 [1.124, 2.948]; *p* = 0.015) and ex-drinker (adjusted OR 0.254 [0.104, 0.620]; *p* = 0.003) were independent determinants of statin non-adherence.

**Conclusion:** Statin adherence was poor in this study. The findings indicated a significant association between inpatients’ lower necessity beliefs and statin non-adherence. More attention should be focused on statin non-adherence in China. Nurses and pharmacists could play an important role in patient education and patient counseling in order to improve medication adherence.

## Introduction

Cardiovascular disease remains the top cause of death in China (Liu et al., 2019). Statins, as a class of medications, have a critical role in the prevention and treatment of cardiovascular diseases. However, it was indicated that only about 1.4% of 0.5 million participants reported current use of statins for the secondary prevention of cardiovascular disease in China (Chen et al., 2014). Although the beneficial effects of statin therapy had been documented in the past 30 years, statin adherence remained suboptimal in clinical practice (Gomez Sandoval et al., 2011; Rodriguez et al., 2019). Around 40%–75% patients discontinued their statin therapy within 1 year after initiation (Banach et al., 2016). Poor adherence limited the efficacy of statin therapy (Xu et al., 2017). Non-adherence or discontinuation of statin therapy was associated with increased risk for cardiovascular and cerebrovascular morbidity, events, and mortality, which significantly increased medical costs (Gomez Sandoval et al., 2011; Li and Huang, 2015; Xu et al., 2017; Rodriguez et al., 2019). Statin adherence was also suboptimal in China. It was reported that 59.2% of patients were of poor statin adherence in Taiwan (Li and Huang, 2015), only 5.4% of 99,655 patients were deemed adherent among new statins users for primary prevention of cardiovascular disease during the initial 12-month follow-up period in Tianjin (Zhao et al., 2020).

Adherence to medications is defined as the process by which patients take their medications as prescribed. Non-adherence to medications can thus occur in the following situations or combinations thereof: late or non-initiation of the prescribed treatment, sub-optimal implementation of the dosing regimen or early discontinuation of the treatment (Vrijens et al., 2012). Many factors may affect medication adherence. Patients’ beliefs and attitude regarding medications were known to be common cause of medication non-adherence (Horne and Weinman, 1999; Horne et al., 2013). The Beliefs about Medicines Questionnaire (BMQ) is a useful tool to identify patients at risk of non-adherence (Horne and Weinman, 1999; Wei et al., 2017). We derive two testable hypotheses for our empirical study. The first one is that statin adherence is poor in the Northwestern China. The second is that patients who have doubts about the necessity of statin and concerns about the potential adverse consequences of statin is more likely to be non-adherent.

Medication adherence is particularly important for positive health outcomes. However, adherence patterns among statin users have not been comprehensively reviewed in the Northwestern China. Scant data are available on the possible association between patients’ beliefs and statin non-adherence among adult Chinese patients. Barriers to medication adherence have to be understood to establish strategies to achieve therapeutic goals (Brown and Bussell, 2011). The objectives of this study are to assess the prevalence of statin non-adherence, and to identify the factors associated with statin non-adherence, especially the associations between inpatients’ beliefs about statins and non-adherence.

## Materials and methods

### Study design and setting

A questionnaire was constructed and conceptualized based on a literature review. This cross-sectional survey was carried out in the department of cardiology and neurology of Xi’an People’s Hospital (Xi’an Fourth Hospital) between February and June 2022. This tertiary hospital is located in Shaanxi Province of Northwestern China. It has around 1,300 beds in all and covers two districts, including 60-bed cardiology unit and 90-bed neurology unit. All the investigators had received standardized training on survey procedures and communication skills.

### Study population and sample size

The inclusion criteria for participants were inpatients who 1) aged ≥18 years; 2) were diagnosed with hyperlipidemia, atherosclerosis, coronary atherosclerotic heart disease, acute coronary syndrome or prior stroke; 3) were prescribed statins (atorvastatin, simvastatin or rosuvastatin); 4) agreed to participate in the survey. It should be noted that the study population comprised not only patients who were started statin treatment during hospitalization but also those who might had been on statin treatment prior to being admitted to the hospital, regardless of when this treatment was initiated. Patients were excluded if they were too ill to participate. The exclusion criteria were inpatients who 1) had been admitted to the ICU or transferred from or to the ICU halfway; 2) experienced adverse clinical outcomes including myocardial infarction, acute cerebral infarction or death during hospitalization; 3) could not communicate due to physical or mental problems.

The minimum number of participants was calculated by using the following formula: *n* = *z* ^2^ *p(1-p)/d* ^2^, where *n* was the sample size, *z* was coefficient of confidence interval (1.96), *p* was prevalence rate, and *d* was type I error level of 0.05. Adherence to long-term therapy for chronic conditions was assumed to be 50% based on previous study (Brown and Bussell, 2011; Nieuwlaat et al., 2014). A minimum sample size of 384 inpatients were required based on the above assumptions. Finally, 524 inpatients were recruited in our study.

### Survey procedures

Content and validity of the original version of the questionnaire was established by an expert panel of the multidisciplinary research team (three experienced clinical pharmacists, one director of a hospital pharmacy, one professor majoring in cardiology, one professor majoring in neurology and one epidemiologist). A pilot study involving 30 participants was also conducted. The questionnaire was revised as necessary after gaining the feedback of experts, as a few questions which were hard to understand were modified or removed. Inpatients were approached by investigators in the medical wards. The purpose and content of the study were explained to eligible inpatients and written informed consents were obtained prior to being enrolled in the study. Face-to-face interviews were conducted individually, using paper-and-pencil method lasting approximately 15–20 min. Inpatients completed the questionnaire either by themselves or with help from the investigators. For the illiterate subjects, the investigators explained the meaning of the items of the questionnaire and recorded their responses. Participants returned their questionnaires to investigators immediately after completion in the wards. Investigators checked carefully for any missing information.

### Measurement instruments

Two validated instruments were used: the Adherence to Refills and Medications Scale (ARMS) was used to assess statin adherence. The BMQ-Specific Scale was used to assess patients’ beliefs about statins. The Chinese versions of the ARMS and BMQ-Specific scales were adapted for use in our study after we obtained authorization from the developers of the scales.

### Beliefs about medicines questionnaire-specific (BMQ-specific)

The BMQ-Specific developed by Horne and Weinman (1999) was used to assess patients’ beliefs about the medication prescribed for a particular illness. In brief, it comprised two scales: 1) a five-item treatment necessity scale Specific-Necessity that assessed the patients’ beliefs about the necessity of taking the medication to maintain or improve their health, and 2) a six-item treatment concern scale Specific-Concerns that focused on beliefs about the treatment’s potential adverse consequences (Horne and Weinman, 1999; Horne, Weinman, Hankins, 1999). Respondents must indicate their degree of agreement with each individual statement of the 11 questions on a five-point Likert scale, ranging from 1 (strongly disagree) to 5 (strongly agree). The total necessity scores were divided by 5 and the total concerns scores were divided by 6, respectively, to give a scale score ranging from 1 to 5. Higher score indicated stronger beliefs. Participants were categorized into four groups (high/low necessity and high/low concerns) based on whether they scored above or below the scale midpoint for the Specific-Necessity and Specific-Concerns scales (Horne and Weinman, 1999; Wei et al., 2017). The previous study suggested that the Chinese version of the BMQ-Specific could serve as a reliable and valid tool for assessing medication beliefs in Chinese patients (Nie et al., 2019). The internal consistency reliability of the BMQ-Specific scale was evaluated using Cronbach’s α coefficient in Chinese population, which indicated a high level of reliability, with α values of 0.784 for necessity and 0.698 for concern subscales, respectively. In addition, the test-retest reliability of the BMQ-Specific scale was evaluated using the intraclass correlation coefficient (ICC), which demonstrated satisfactory reliability and stability (ICC = 0.759). Furthermore, the ratio of χ^2^ to degrees of freedom (df) was 2.231, and the Goodness of Fit Index (GFI) was 0.928, while the Standardized Root Mean Square Residual (SRMR) and Root Mean Square Error of Approximation (RMSEA) were 0.074 and 0.075, respectively, indicating a good validity (Cai et al., 2020). The BMQ-Specific scale was provided as a supplementary file (see Supplementary File S1).

### Medication adherence

The ARMS was developed to evaluate self-reported adherence to taking and refilling medications among patients with chronic disease (Kripalani et al., 2009; Kripalani et al., 2015). The ARMS scale comprised two subscales: eight items designed to assess adherence to taking medications and four items designed to refill prescriptions, respectively. A 4-point Likert-type scale was used to score responses as “none,” “some of the time,” “most of the time,” and “all of the time,” assuming the values from 1 to 4, respectively. Lower score, ranging from 12 to 48, represented better adherence. According to the published literature (Kripalani et al., 2009; Polanski et al., 2020), participants were classified into two groups based on their total adherence score: <16 points (adherence group) and ≥16 points (non-adherence group), respectively. The Chinese version of the ARMS scale was found to be reliable and valid for assessing medication adherence of Chinese patients with chronic disease. The internal consistency of the ARMS scale was evaluated using Cronbach’s α coefficient, which indicated a high level of reliability (*α* = 0.731). The test-retest reliability of the ARMS scale was assessed using Spearman’s rho, which indicated satisfactory reproducibility and stability (rho = 0.871). Moreover, the criterion validity of the ARMS scale was assessed using Spearman’s rho, which demonstrated satisfactory validity (rho = 0.711) (Wu et al., 2021). The ARMS scale was provided as a supplementary file (see Supplementary File S2).

### Data collection

The designed questionnaire included sociodemographic, clinical data, the ARMS scale, the BMQ-Specific scale, and other information. Sociodemographic characteristics included age, gender, body height, body weight, smoking status, alcohol consumption, occupational status, place of residence, marital status, and education level. Information about diagnosis at admission, comorbidity conditions, health insurance, statin prescribed, and co-medications potentially influencing patients’ adherence to statin were collected from the electronic medical records. Co-medications included anticoagulants, antiplatelets, antihypertensives, hypoglycemics and lipid-lowing agents (except statins). To assess patient-related factors associated with statin non-adherence, we collected the duration of statin treatment, patients’ awareness of the primary reason for prescription of statins, regular review, and regular exercise per week. The questionnaire adopted in our study was provided as a supplementary file (see Supplementary File S3). Three branded and generic statin preparations including atorvastatin, rosuvastatin and simvastatin were available in our hospital when this study was conducted.

### Outcome measurements

The primary outcome was the prevalence of statin non-adherence. Factors associated with statin non-adherence were investigated as the second outcome in our study, and the association between inpatients’ beliefs about statins and non-adherence.

### Statistical analysis

Basic characteristics were presented using frequencies (percentages) for categorical variables. Differences in demographic and clinical characteristics between adherent and non-adherent inpatients were evaluated using the Chi-square test for categorical variables, the Mann-Whitney test for skew continuous variables, and the independent sample *t*-test for normal continuous variables. Variables found to be significant at *p*-value < 0.1 from the univariable logistic regression were included in multivariable logistic regression model to characterize the independent factors associated with statin non-adherence. Receiver operator characteristic (ROC) analysis was conducted to assess the performance of the logistic regression model in predicting statin non-adherent. All analysis were performed by using the SPSS V25.0 Statistical Software Package for Windows. A *p*-value < 0.05 was considered statistically significant for all analyses.

## Results

A total of 550 respondents agreed to participate in the survey. Twelve participants did not return the questionnaire and 14 questionnaires were uncompleted, 524 (95.3%) respondents were included in our study.

The mean age of the 524 participants was 63.0 ± 12.3 years, and the majority (64.7%) were male. The demographic and clinical characteristics of the study subjects were presented in Table 1. A total of 426 (81.3%) patients were non-adherent to statin. All inpatients were prescribed statin monotherapy during the study period. Atorvastatin was taken by 52.7%, rosuvastatin by 44.1% and simvastatin by 3.2% of the study population. Of the 524 inpatients, 229 (43.7%) inpatients expressed strong beliefs about the treatment necessity, while 246 (47.0%) inpatients expressed strong concerns about the potential negative effects.

**TABLE 1:** Demographic and clinical characteristics of the study subjects.

Characteristics	Overall population (*n* = 524, %)	Adherent (*n* = 98, %)	Non-adherent (*n* = 426, %)	*p-value*
Age (years)				0.544
≤44	39	4(10.3)	35(89.7)	
45–54	81	17(21.0)	64(79.0)	
55–64	162	31(19.1)	131(80.9)	
65–74	148	31(20.9)	117(79.1)	
≥75	94	15(16.0)	79(84.0)	
BMI(kg/m^2^)				0.986
<18.5	17	3(17.6)	14(82.4)	
18.5–23.9	173	31(17.9)	142(82.1)	
24–27.9	243	47(19.3)	196(80.7)	
≥28	91	17(18.7)	74(81.3)	
Gender				0.542
Female	185	32(17.3)	153(82.7)	
Male	339	66(19.5)	273(80.5)	
Smoking status				0.818
Non-smoker	308	59(19.2)	249(80.8)	
Current smoker	188	35(18.6)	153(81.4)	
Ex-smoker	28	4(14.3)	24(85.7)	
Alcohol consumption				0.003*
Non-drinker	421	71(16.9)	350(83.1)	
Current drinker	81	17(21.0)	64(79.0)	
Ex-drinker	22	10(45.5)	12(54.5)	
Diagnosis				
Hyperlipidemia	28	5(17.9)	23(82.1)	0.906
Atherosclerosis	3	2(66.7)	1(33.3)	0.091
Coronary atherosclerotic heart disease	381	68(17.8)	313(82.2)	0.413
Acute coronary syndrome	141	33(23.4)	108(76.6)	0.094
Prior stroke	47	5(10.6)	42(89.4)	0.137
Duration of statin treatment				0.526
<1 year	307	57(18.6)	250(81.4)	
1–5 years	151	29(19.2)	122(80.8)	
6–9 years	35	4(11.4)	31(88.6)	
≥10 years	31	8(25.8)	23(74.2)	
Occupational status				0.797
Employed	296	55(18.6)	241(81.4)	
Unemployed	7	2(28.6)	5(71.4)	
Retired	221	41(18.6)	180(81.4)	
Residence				0.809
Rural	241	44(18.3)	197(81.7)	
Urban	283	54(19.1)	229(80.9)	
Health insurance				0.923
Uninsured	137	26(19.0)	111(81.0)	
Insured	387	72(18.6)	315(81.4)	
Marital status				0.798
Single/Unmarried	3	1(33.3)	2(66.7)	
Married and living with a partner	489	90(18.4)	399(81.6)	
Divorced or widowed	32	7(21.9)	25(78.1)	
Education level				0.412
≤High school graduation	423	82(19.4)	341(80.6)	
≥University (college) graduation	101	16(15.8)	85(84.2)	
Comorbidity conditions				
Hypertension	266	49(18.4)	217(81.6)	0.911
Diabetes mellitus	117	17(14.5)	100(85.5)	0.226
Atrial fibrillation	91	15(16.5)	76(83.5)	0.561
Statin prescribed				0.023*
Atorvastatin	276	63(22.8)	213(77.2)	
Rosuvastatin	231	31(13.4)	200(86.6)	
Simvastatin	17	4(23.5)	13(76.5)	
Concurrent used drugs				
Anticoagulants	36	7(19.4)	29(80.6)	0.906
Antiplatelets	479	91(19.0)	388(81.0)	0.571
Antihypertensives	138	25(18.1)	113(81.9)	0.837
Hypoglycemics	103	14(13.6)	89(86.4)	0.138
Lipid-lowering agents (except statins)	17	3(17.6)	14(82.4)	0.910
Patients’ awareness of the primary reason for prescription of statins				0.722
No	233	42(18.0)	191(82.0)	
Yes	291	56(19.2)	235(80.8)	
Necessity beliefs				0.038*
Low	295	46(15.6)	249(84.4)	
High	229	52(22.7)	177(77.3)	
Concerns beliefs				0.261
Low	278	57(20.5)	221(79.5)	
High	246	41(16.7)	205(83.3)	
Regular review				0.920
No	281	53(18.9)	228(81.1)	
Yes	243	45(18.5)	198(81.5)	
Regular exercise per week				0.043*
<3 times	246	37(15.0)	209(85.0)	
≥3 times	278	61(21.9)	217(78.1)	

The data are presented as numbers (proportions). Bold values indicate a *p*-value <0.05. BMI, body mass index.

Univariable and multivariable logistic regression analysis of factors associated with statin non-adherence were provided in Table 2.

**TABLE 2:** Univariable and multivariable logistic regression analysis of factors associated with statin non-adherence.

Characteristics	Unadjusted OR (95% CI)	*p-value*	Adjusted OR (95% CI)	*p-value*
Age (years)				
≤44	1.000(Reference)			
45–54	0.430(0.134–1.379)	0.156		
55–64	0.483(0.160–1.460)	0.197		
65–74	0.431(0.142–1.306)	0.137		
≥75	0.602(0.186–1.944)	0.396		
BMI(kg/m^2^)				
18.5–23.9	1.000(Reference)			
<18.5	1.019(0.276–3.761)	0.978		
24–27.9	0.910(0.551–1.504)	0.714		
≥28	0.950(0.494–1.829)	0.879		
Gender				
Female	1.000(Reference)			
Male	0.865(0.543–1.379)	0.543		
Smoking status				
Non-smoker	1.000(Reference)			
Current smoker	1.036(0.651–1.648)	0.882		
Ex-smoker	1.422(0.475–4.253)	0.529		
Alcohol consumption				
Non-drinker	1.000(Reference)		1.000(Reference)	
Current drinker	0.764(0.422–1.381)	0.373	0.773(0.420–1.421)	0.407
Ex-drinker	0.243(0.101–0.585)	0.002*	0.254(0.104–0.620)	0.003*
Diagnosis				
Hyperlipidemia	1.223(0.403–3.714)	0.722		
Atherosclerosis	0.094(0.008–1.171)	0.066	0.135 (0.012–1.567)	0.109
Coronary atherosclerotic heart disease	0.741(0.329–1.669)	0.469		
Acute coronary syndrome	0.530(0.239–1.177)	0.119		
Prior stroke	1.854(0.709–4.852)	0.208		
Duration of statin treatment				
<1 year	1.000(Reference)			
1–5 years	0.959(0.584–1.576)	0.869		
6–9 years	1.767(0.600–5.205)	0.302		
≥10 years	0.656(0.279–1.540)	0.333		
Occupational status				
Employed	1.000(Reference)			
Unemployed	0.571(0.108–3.018)	0.509		
Retired	1.002(0.640–1.568)	0.993		
Residence				
Rural	1.000(Reference)			
Urban	0.947(0.609–1.473)	0.809		
Health insurance				
Uninsured	1.000(Reference)			
Insured	1.025(0.623–1.686)	0.923		
Marital status				
Married and living with a partner	1.000(Reference)			
Single/Unmarried	0.451 (0.040–5.030)	0.518		
Divorced or widowed	0.806(0.338–1.920)	0.626		
Education level				
≤High school graduation	1.000(Reference)			
≥University (college) graduation	1.277(0.711–2.295)	0.413		
Comorbidity conditions				
Hypertension	0.967(0.617–1.515)	0.882		
Diabetes mellitus	1.500(0.838–2.685)	0.172		
Atrial fibrillation	1.249(0.681–2.291)	0.473		
Statin prescribed				
Atorvastatin	1.000(Reference)		1.000(Reference)	
Rosuvastatin	1.908(1.191–3.057)	0.007*	1.820(1.124–2.948)	0.015*
Simvastatin	0.961(0.303–3.052)	0.947	0.975(0.280–3.397)	0.968
Concurrent used drugs				
Anticoagulants	0.639(0.194–2.106)	0.461		
Antiplatelets	0.551(0.171–1.776)	0.318		
Antihypertensives	0.956(0.567–1.612)	0.867		
Hypoglycemics	1.642(0.876–3.077)	0.122		
Lipid-lowing agents (except statins)	1.008(0.276–3.687)	0.990		
Patients’ awareness of the primary reason for prescription of statins				
No	1.000(Reference)			
Yes	0.923(0.592–1.438)	0.722		
Necessity beliefs				
High	1.000(Reference)		1.000(Reference)	
Low	1.590(1.023–2.472)	0.039*	1.607(1.019–2.532)	0.041*
Concerns beliefs				
High	1.000(Reference)			
Low	0.775(0.497–1.209)	0.262		
Regular review				
No	1.000(Reference)			
Yes	1.023(0.658–1.589)	0.920		
Regular exercise per week				
<3 times	1.000(Reference)		1.000(Reference)	
≥3 times	0.630(0.401–0.988)	0.044*	0.665(0.418–1.059)	0.085

Bold values indicated a *p*-value <0.05. OR, odds ratio; CI, confidence interval.

In the univariable analysis, four factors were significantly associated with statin non-adherence: necessity beliefs (*p* = 0.039), statin prescribed (*p* = 0.007), alcohol consumption (*p* = 0.002), regular exercise per week (*p* = 0.044). Patients reported low necessity beliefs about statins were less likely to be adherent compared with those reported high necessity beliefs (unadjusted OR 1.590 [1.023–2.472]). Patients who were prescribed rosuvastatin were less likely to be adherent compared with those prescribed atorvastatin (unadjusted OR 1.908 [1.191–3.057]). Ex-drinkers indicated higher odds of statin adherence compared with non-drinkers (unadjusted OR 0.243 [0.101–0.585]). Patients who exercised more than 3 times regularly per week were more likely to be adherent to statin therapy compared with patients who exercised less than three times (unadjusted OR 0.630 [0.401, 0.988]).

In the multivariable logistic regression analysis (adjusted by alcohol consumption, atherosclerosis, atherosclerosis, statin prescribed, patients’ necessity beliefs about statins, regular exercise per week), patients’ low necessity beliefs about statins (adjusted odds ratio [OR] and 95% confidence interval [CI], 1.607 [1.019, 2.532]; *p* = 0.041) and prescribed rosuvastatin (adjusted OR 1.820 [1.124, 2.948]; *p* = 0.015) were associated with lower odds of statin adherence while ex-drinker (adjusted OR 0.254 [0.104, 0.620]; *p* = 0.003) was associated with higher odds of statin adherence.

**FIGURE 1:**
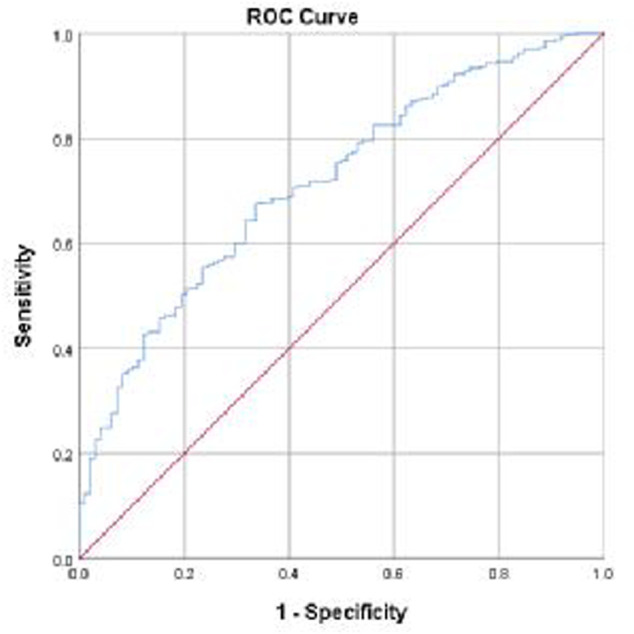
Receiver Operating Characteristic (ROC) curve for logistic regression model predicting statin non-adherent: AUC of ROC curve = 0.716 (95% CI = 0.663–0.770). The ROC curve was produced in SPSS.

The ROC curve for logistic regression model predicting statin non-adherent was shown in Figure 1. The model provided an area under the curve (AUC) for the ROC curve of 0.72 (95% CI = 0.66–0.77).

## Discussion

Medication adherence has been defined as the extent to which a patient takes medications as prescribed by their healthcare providers (Osterberg and Blaschke, 2005). Based on previously published studies, medication adherence varies between 32% and 79% for statins users (Huiskes et al., 2021). A total of 426 (81.3%) patients were non-adherent to statin in our study, therefore, the adherent rate was only 18.7%. Statin adherence in our study was substantially lower than the results found in developed countries such as Netherlands (Huiskes et al., 2021), Republic of Korea (Chung et al., 2018), Finland (Rannanheimo et al., 2015) and United States (Chan et al., 2010), as well as other regions of China (Li and Huang, 2015; Zhang et al., 2022). It was reported that only 5.4% of 99,655 patients were deemed adherent among new statin users for primary prevention of cardiovascular disease during the initial 12-month follow-up period in Tianjin, which showed lower adherence level than our study (Zhao et al., 2020). It was unexpected that statin adherence was so poor in our study. The reasons may include that there was no recognized gold-standard method to measure adherence. Different definitions and measures of medication adherence contributed to the variations of adherence level among studies and population groups (Chung et al., 2018). The variable medication adherence levels among countries might also be due to different healthcare delivery systems (Osterberg and Blaschke, 2005; Bushnell et al., 2011). Literature had indicated patients with adequate medication literacy were more likely to be adherent (Zheng et al., 2020). However, the level of medication literacy among patients with coronary heart disease was suboptimal in China and needed to be improved (Zheng et al., 2020). Furthermore, new statin users were more likely to be non-adherent compared with previous users (Kopjar et al., 2003). It was reported that the first 180 days of follow-up was the most critical period when many patients became non-adherent or discontinued treatment (Ofori-Asenso et al., 2018). It appeared that 48.2% of the patients were non-adherent among new statin users, and 23.9% discontinued within the first treatment year (Ofori-Asenso et al., 2018). A total of 307 (58.6%) inpatients in our study commenced statin therapy in the past year, which might contribute to poor statin adherence in our study as well.

Both unadjusted and adjusted results revealed that low necessity beliefs were significantly associated with non-adherence. Our findings were consistent with previous findings that the odds of non-adherence were significantly increased when patients reported low necessity beliefs (Horne and Weinman, 1999; Horne et al., 2013; Foot et al., 2016; Huiskes et al., 2021). Study had mentioned that illness perception was an underlying factor for beliefs about the treatment necessity (Horne, Weinman, Hankins, 1999). The higher perception of illness was associated with increased likelihood of stronger agreement on the necessity of treatment, as well as better adherence to the therapy (Chung et al., 2018; Cai et al., 2020). Stronger beliefs in the necessity of the medication occurred in patients who believed their illness to be lasting or experienced more symptoms (Horne, Weinman, Hankins, 1999). Patients with asymptomatic diseases who did not realize the need to take medicine were more likely to be non-adherent (Xu et al., 2020). Low adherence may be a choice between patients’ assessment of their personal treatment needs and their concerns about the potential adverse consequences of taking medicine (Cai et al., 2020). Our analysis found Specific-Concerns was not associated with non-adherence. Patients’ awareness of the primary reason of statin therapy was also not found to be associated with non-adherence in our study. Further investigation is required to measure the association between patients’ beliefs and their adherence.

Atorvastatin was the most frequently prescribed statin in our study, which was consistent with previous study (Hsieh et al., 2017). Inpatients who took rosuvastatin during the study period had lower adherence than atorvastatin. Limited literature had revealed direct association between types of statins and adherence. One study revealed that patients were more adherent to atorvastatin compared with other statin preparations (Xie et al., 2022), which was in accordance with our study. Another study found that patients prescribed atorvastatin or rosuvastatin indicated higher odds of statin adherence compared with those prescribed simvastatin (Morotti et al., 2019). It was also reported that the persistence was higher with atorvastatin compared with simvastatin (Huser et al., 2005). Higher likelihood of adherence to atorvastatin might be due to its better tolerability, efficacy and safety (Xie et al., 2022).

There were conflicting data regarding the association between alcohol consumption and medication adherence. It was reported that alcohol consumption was associated with increased risk for medication non-adherence (Bryson et al., 2008). However, a study in Republic of Korea revealed that ex-drinkers were less adherent to statins than drinkers (Chung et al., 2018). Our study suggested that ex-drinkers indicated higher odds of statin adherence compared with non-drinkers. In the univariable analysis, patients who exercised more than three times regularly per week were more likely to be adherent to statin therapy compared with patients who exercised less than three times. The finding in our study was contrary to previous study, which revealed that regular exercise per week was not associated with adherence (Chung et al., 2018). These findings might be explained by the fact that patients with healthy lifestyle might adherent because they were more likely to seek for healthier behaviors (Brookhart et al., 2007).

Poor adherence limited the efficacy of statin therapy, which might trigger risk of cardiovascular and cerebrovascular adverse events (Gomez Sandoval et al., 2011; Xu et al., 2017). A variety of effective interventions were recommended to improve medication adherence (Bates et al., 2009; Gatwood and Bailey, 2014). Compared with the demographic and clinical factors associated with non-adherence, patients’ beliefs were more readily modifiable (Clifford et al., 2006). Optimal adherence to medications could be supported by taking account of patients’ necessity beliefs and concerns (Horne et al., 2013). More effective communication with patients is crucial to emphasize the importance of continuous statins therapy even under the conditions of asymptomatic, and make them aware of the potential risk of adverse health outcomes (Maningat et al., 2013; Banach et al., 2016; Kruger et al., 2018; Tan et al., 2019). However, clinicians were required to meet more patients in less time, which made it was difficult to perform enough communication with patients (Nieuwlaat et al., 2014). Study have revealed insufficient communication between patients and their doctors regarding the prescription, and 32% overall and 24% of patients with 3 or more chronic conditions reported no dialogue with their doctor about all their medicines in the last 12 months (Wilson et al., 2007). The lack of adequate explanation about the diseases as well as the benefits and potential side effects of medication provided by the clinicians were acknowledged as strong contributors to non-adherence (Devaraj et al., 2017; Brinton, 2018). Many patients discontinued statin use because of uncertainty about the benefits of statins and concerns about adverse effects (Fung et al., 2010). Considering that clinicians have limited time, nurses or pharmacists-led education programs and reminder systems have been shown to be active interventions to improve statin adherence (De Geest and Sabate, 2003; Bates et al., 2009; Marzec and Maddox, 2013; Gatwood and Bailey, 2014; Nieuwlaat et al., 2014). A better assessment of the patients’ needs and barriers to medication adherence could be performed through face-to-face education. Education augments the health literacy of patients and improves medication adherence by increasing the knowledge of their conditions, complications and management (Tan et al., 2019). Pharmacist-led counseling program on medication adherence for patients helped establishing a routine of daily self-medication and potentially improved their long-term clinical outcomes (Taitel et al., 2012).

## Strengths and limitations

This is the first study to assess patients’ adherence to statins and explore the association between beliefs about medicines and self-reported adherence in the Northwestern China. A better understanding of the prevalence of statin non-adherence and barriers to statins adherence is critical for designing effective interventions to improve adherence. We believe that this study will help healthcare providers understand that non-adherence to statins is a serious problem for patients. Inpatients who had stronger doubts about their personal need for statins were significantly more likely to be non-adherent. Our findings provide a basis for future accessible and systematic interventions to improve medication adherence in China. Our study has several limitations. First, it was conducted in one hospital, which could not represent the general situation in China. Prospective designs in a wide range of settings are necessary for a thorough assessment of the role of beliefs in predicting non-adherence. The second one is that adherence was only measured by a self-reported questionnaire in this study. Although the ARMS Scale has been validated as a measure of general behavior in chronic diseases, self-reported adherence may not be the best measurement for medicine adherence because of subjective and sensitive to social desirability bias (Huiskes et al., 2021). Third, the majority of patients in our study were elder people with multiple comorbidities. As potential factors affecting medication adherence, polypharmacy, health literacy, drug side effects and the price or out-of-pocket to obtain statins were not investigated in the current study, these could be the reasons for poor adherence in our study. In addition, there were differences in adherence to statin therapy between new users and previous users. Inpatients were not stratified according to the duration of statin treatment, which might lead to bias. Further prospective research is required to confirm the factors associated with statin adherence.

## Conclusion and implications

Statin adherence was poor in the Northwestern China. This study indicated a significant association between patients’ lower necessity beliefs and statin non-adherence. More attention should be focused on statin non-adherence in China. In addition, our results also suggested that groups of individuals who were prescribed rosuvastatin were less adherent, and patients who were ex-drinker might adherent. Nurses and pharmacists could play an important role in patient education and patient counseling in order to improve medication adherence.

## Data Availability

The original contributions presented in the study are included in the article/Supplementary Material, further inquiries can be directed to the corresponding authors.
