# Supplementary material for: Relationships between beliefs about statins and non-adherence in inpatients from Northwestern China: a cross-sectional survey
Source: Front Pharmacol. 2023 Jun 9;14:1078215. doi: 10.3389/fphar.2023.1078215 (PMC10289550; doi:10.3389/fphar.2023.1078215)
Supplement: Supplementary file 2 [file DataSheet1.doc]

| **YOUR VIEWS ABOUT** |
| --- |
| **MEDICINES PRESCRIBED FOR YOU** |
| **(BMQ SPECIFIC)** |

We would like to ask you about your personal views about medicines prescribed for you.

These are statements other people have made about their medicines.

Please show how much you agree or disagree with them by ticking the appropriate box.

| **There are no right or wrong answers.** |
| --- |
| **We are interested in your personal views** |

|  | **Views about MEDICINES PRESCRIBED FOR YOU:** | **Scale Structure** |
| --- | --- | --- |
| BS1 | My health, at present, depends on my medicines N | N= Specific Necessity |
| BS2 | Having to take medicines worries me C | C= Specific Concerns |
| BS3 | My life would be impossible without my medicines N |  |
| BS5 | I sometimes worry about long-term effects of my medicines C |  |
| BS4 | Without my medicines I would be very ill N |  |
| BS6 | My medicines are a mystery to me C |  |
| BS7 | My health in the future will depend on my medicines N |  |
| BS8 | My medicines disrupt my life C |  |
| BS9 | I sometimes worry about becoming too dependent on my medicines C |  |
| BS10 | My medicines protect me from becoming worse N |  |
| BS11 | These medicine give me unpleasant side effects C |  |

All items scored: 5= strongly agree, 4= agree, 3= uncertain, 2= disagree,

1= strongly disagree

**FORMULA FOR COMPUTING BMQ SPECIFIC SCALES**

**5 item Sepcific Necessity scale**

- sum of scores on all N items. Divide by 5 (number of items in scale) to

give a scale score (range 1-5)

**6-item Specific Concerns**

- sum of scores on all N items. Divide by 6 (number of items in scale) to

give a scale score (range 1-5)

SCORING INSTRUCTIONS BMQ-Specific 11 _04 (3) © Professor Rob Horne 1999
